# An Entangled Pedagogy: Looking Beyond the Pedagogy—Technology Dichotomy

**DOI:** 10.1007/s42438-022-00302-7

**Published:** 2022-04-02

**Authors:** Tim Fawns

**Affiliations:** Edinburgh Medical School, University of Edinburgh, 49 Little France Crescent, Edinburgh, EH16 4SB UK

## Abstract

‘Pedagogy first’ has become a mantra for educators, supported by the metaphor of the ‘pedagogical horse’ driving the ‘technological cart’. Yet putting technology first *or* last separates it from pedagogy, making us susceptible to technological or pedagogical determinism (i.e. where technology is seen either as the driving force of change or as a set of neutral tools). In this paper, I present a model of entangled pedagogy that encapsulates the mutual shaping of technology, teaching methods, purposes, values and context. Entangled pedagogy is collective, and agency is negotiated between teachers, students and other stakeholders. Outcomes are contingent on complex relations and cannot be determined in advance. I then outline an aspirational view of how teachers, students and others can collaborate whilst embracing uncertainty, imperfection, openness and honesty, and developing pedagogical knowledge that is collective, responsive and ethical. Finally, I discuss implications for evaluation and research, arguing that we must look beyond isolated ideas of technologies or teaching methods, to the situated, entangled combinations of diverse elements involved in educational activity.

## Introduction: Moving Past the Technology—Pedagogy Dichotomy

‘Pedagogy first’ has become a mantra against worries that technology might overly influence education (Cousin 2005; Tsui and Tavares 2021). In response to the hype and hyperbole of marketing and research discourse, where each new technology inevitably transforms or enhances learning (Kirkwood and Price 2012), many educators want the ‘pedagogical horse’ to drive the ‘technological cart’ (Sankey 2020). Being ‘pedagogically driven’ (Anderson and Dron 2011) offers reassurance that ‘nothing is changing in a context in which rather a lot is changing’ (Brett and Cousin 2010: 610). However, whilst it is certainly problematic to introduce technology without sufficient consideration of the aims or established practices of teachers and students (i.e. a ‘technology first’ approach), attempting to put technology last leaves educators susceptible to an inadequate appreciation of complexity relating to how it is entangled in educational activity.

Both technology-led and pedagogy-led positions decontextualise technology and make us vulnerable to different forms of determinism (see Oliver 2011 for a review). Most relevant to this paper are technological and pedagogical determinism. Technological determinism sees technology as driving social change (Kaplan 2009), where outcomes can be predicted by design and functionality. This view can be optimistic (e.g. technology inevitably leads to greater efficiency) or pessimistic (e.g. technology inevitably dehumanises or harms us) (Chandler 1995; Friesen 2008; Kanuka 2008). Moral disapproval often awaits those who oppose, or impose, technology, respectively (Chandler 1995). A ‘pedagogy first’ position could suggest pessimistic technological determinism, or it could suggest pedagogical determinism (see also *use determinism,* Kanuka 2008, and *human determinism*, Berg 1998) where people (e.g. teachers) drive change, using methods and technology to achieve their objectives (Anderson and Dron 2011). In pedagogical determinism, pedagogy is attributed with unassailable, decontextualised characteristics (Berg 1998) and technology’s influence on thinking and practising is neglected (Chandler 1995; Kanuka 2008).

Whilst technology is sometimes imposed before course-level decisions are made about what should be done with it (e.g. centralised virtual learning environments [VLEs] or learning analytics dashboards), the greater problem may be where teachers themselves start with a method before sufficiently considering their own or their students’ purposes, values and contexts. Choices about technology, tasks, social configurations and resources are then restricted by what is possible within an already-constrained conception of teaching. For example, emergency remote teaching (Hodges et al. 2020), during the COVID-19 pandemic, showed that traditional methods and attempts to ‘simulate physical classroom teaching’ (Tsui and Tavares 2021) can reinforce practices unsuitable to online contexts. Primacy of methods can suggest technological determinism, where methods are seen as technologies (see Dron 2021), or pedagogical determinism, where methods are seen as largely independent of technology (Anderson and Dron 2011). The former is exemplified by reductive comparisons of methods (e.g. lectures vs problem-based learning) where teacher and learner agency are marginalised. Clark (1983) gives an example of the latter, arguing that teachers implement methods, and technologies are ‘mere vehicles that deliver instruction but do not influence student achievement’ (p. 445). The form of determinism relates to who or what is conceived of as the driving force (e.g. the method itself or the teacher who employs it).

Determinism is appealing because it suggests simple possibilities for solving complex problems. Oliver (2011) contrasts ‘hard’ determinism (where technologies cause inevitable social change, or where humans have complete autonomy) with ‘soft’ determinism that attributes causal power to technology or humans but acknowledges other forces and social, cultural and historical relations. A general conclusion from the philosophy of technology literature is that extremes of any of these views are problematic, and we should consider the influence of technology as part of a complex set of wider relations (Chandler 1995). Technology, users and social context all matter, and all partially determine activity (Winner 1980).

Avoiding determinism requires a holistic view of situated, purposeful uses of technology (Berg 1998; Oliver 2011). Sociomaterial approaches to the study of technology, including cultural historical activity theory (Engeström and Sannino 2010), practice theory (Nicolini 2013; Schatzki et al. 2001), actor network theory (Latour 2005) and posthumanism (Barad 2007), can help us navigate the territory between technology-led and pedagogy-led views through a strong commitment to focusing on relations rather than elements (e.g. for Barad 2007, individuals and objects do not exist at all except as things always already entangled in activity). Relatedly, postdigital views see all digital activity as social, material and embedded in rich and diverse contexts (Fawns 2019; Jandrić et al. 2018). Taking neither individual teachers nor technology, as the unit of analysis, but a holistic view of entangled elements, provides a stronger basis for taking complexity into account (Edwards 2010; Fenwick 2015). The entangled pedagogy model, presented next, draws from sociomaterial and postdigital perspectives to outline the key relations within educational practice.

## A Model of an Entangled Pedagogy

### Illusions Vs Actuality

**Fig. 1:**
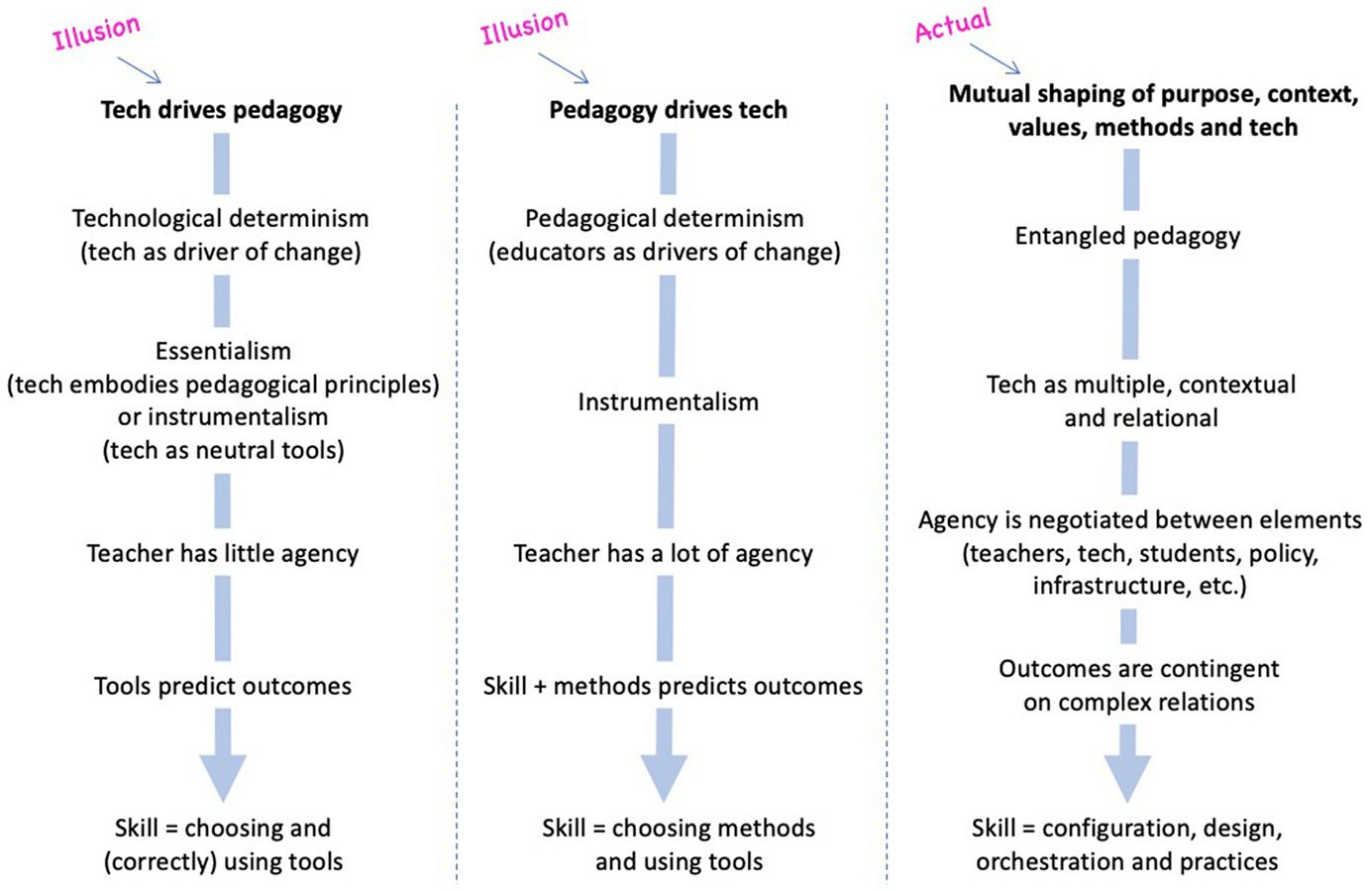
An entangled pedagogy: views of the relationship between technology and pedagogy

Figure 1, below, shows three views of the relationship between technology and pedagogy. Column 1 represents a ‘technology first’ view, in which technology is seen as the driver of educational activity and outcomes. Column 2 represents a ‘pedagogy first’ (or ‘technology last’) view, in which educators are seen as the driving force, and technology is subservient to the teaching approach. Whilst it is possible to choose technologies before deciding what to do with them (or why), and whilst it is also possible to choose teaching approaches before thinking about technology, Columns 1 and 2 are labelled as illusions because they portray what follows these choices as independent of other factors. Each misinterprets educational situations and suggests an unrealistic level of control and predictability (Chandler 1995). *Actual* educational activity is always a complex entanglement of factors, iteratively and mutually shaping each other. Column 3 represents an entangled view, in which pedagogy is constituted not just by methods and technology, but also the purposes, contexts and values of teachers, students and other stakeholders. Whilst technology does have implications for practice, and teachers should aim to make good use of its educational possibilities (Bates 2019), these possibilities are socially and materially situated, and relate to the traditions, practices, culture, policy and infrastructure in which they are embedded (Fawns 2019).

Column 1’s illusion is promoted by marketing and research discourse that conveys technological determinism as self-evident, natural or common sense (Chandler 1995; Feenberg 2001). Examples include claims about universal qualities or impacts of online learning (e.g. Zimmerman 2020), attributions of outcomes or student experiences to technological platforms (Aitken and Hayes 2021), descriptions of students as ‘digital natives’ (Jones 2015; Oliver 2011) or complaints about teachers unable to adapt to the ‘digital age’ (Clegg 2011). Column 2’s illusion is promoted by educator networks, for example, through presentations and blog posts endorsing ‘pedagogy first’ as a common-sense, human-led approach (e.g. Ash-Brown 2020; Lukes 2019; Sheninger 2016). More subtle indicators of Column 2 include references to ‘tools’ (e.g. *it’s not the tool, it’s how you use it*) or ‘technology-enhanced learning’ (Cousin 2005). ‘Enhancement’ can reassure teachers that technology ‘will only make better what is already good’ (Cousin 2005: 121) but it implies control over a reified, linear idea of learning (Bayne 2015; Kirkwood and Price 2014).

Recognising that technology and pedagogy are, inevitably, entangled, opens up possibilities for more meaningful analyses of educational activity (Cousin 2005). Yet the examples in the previous paragraph show how the negotiation of essentialism, instrumentalism and determinism is nuanced and fraught (Feenberg 1999; Finnegan 1989). Columns 1 and 2 may indicate entrenched assumptions, or temporary and unintentional positions. Avoiding these illusions requires vigilance in attending to situated, emergent combinations of educational factors (Berg 1998). The entangled model is intended as a guide for educators to navigate the illusions of Columns 1 and 2 in their design and practice.

### Pedagogy as Encapsulating Technology, Methods, Contexts, Values and Purposes

Anderson and Dron (2011) use the metaphor of a dance, in which ‘technology sets the beat and creates the music, while the pedagogy defines the moves’ (p. 81). In the entangled model, pedagogy *is* the dance. Methods and technology are just part of the constituent components of any situated enactment of education. Since technology is entangled within pedagogy, it is not possible to first choose a pedagogy and then a technology, nor can pedagogy be tacked onto an existing instantiation of technology. Placing pedagogy above technology does not imply pedagogical determinism because teachers and educational designers have only partial and relational agency. Neither they, nor their methods, can determine outcomes. Teachers may lead the choreography, but they have only limited control over how the dance plays out (Anderson and Dron 2011; Dron 2021; Gravett et al. 2021). Furthermore, teaching, in this model, is not just done by teachers but by a range of stakeholders in a combined, mutual effort (Dron 2021; Fawns et al. 2021a). Students co-configure and co-design as they reinterpret and complete teachers’ plans (Dron 2021; Fawns et al. 2022; Goodyear 2015). Learning technologists and information technology staff procure and configure platforms that enable and constrain local teaching. Administrators influence processes and relationships between teachers and students. Policymakers shape culture and practice. Educational activity is emergent, and the roles of teachers and technologies are entangled within a broader conception of pedagogy, along with methods, purposes, values and context, as outlined below.

#### Technology

Technology is pervasive. Humans have always made and used technology, manipulating objects to achieve day-to-day functions (Chandler 1995; Nye 2006), shape experiences (Nardi 1996) and bring order to the world (Winner 1980). Education is always enacted through technology, and teachers cannot avoid learning to use it (Dron 2021). Some technologies are so embedded in educational systems that they are almost invisible. For example, Murphy and colleagues (2001) suggest that the physical classroom ‘itself is a technology, or comprises a set of technologies which we mostly take for granted—physical materials such as desks and chairs, black, white and green boards, chalk, pens, projection devices, worksheets, textbooks, notebooks, lighting and sound regimes and so on’. (p. 2). Our distinctions between ‘technology-enhanced’ and general learning, or even digital education and general education, are somewhat artificial (Fawns 2019).

The term ‘affordances’ is frequently used to describe what can be done with a particular technology, but it can imply homogenous users and abstracted properties (Oliver 2005; 2011). Technologies are not fixed, homogenous *things* with generalisable characteristics or consequences (Chandler 1995). Two problematic understandings of technology, *essentialism* and *instrumentalism*, share an assumption that we can link predetermined functions with expected practices and outcomes (Hamilton and Friesen 2013). In essentialism, technology is imbued with ‘ideological bias’ (Postman 1993: 13), ‘inalienable qualities’ or intrinsic, abstract pedagogical principles (Hamilton and Friesen 2013) that are independent of human activity. In contrast, instrumentalism sees technology as a set of neutral tools, defined by technical properties, independent of social forces, and ‘subservient to human choices’ (Kaplan 2009: 4). Whilst pedagogical determinism is associated with instrumentalism, a technological determinist view can coincide with either essentialist or an instrumental views (Feenberg 2006). Instrumentalism’s neutrality can mask values of efficiency (Feenberg 2006), where what matters is that a tool works, and what works can be ‘determined objectively according to universally valid, scientifically established principles’ (Kaplan 2009: 4).

In practice, technology is always an assembly of multiple other technologies (Dron 2021), and always more than the sum of its parts (Chandler 1995). It is always entangled in context, and understood differently by different people in different settings (Dron 2021). Rather than focusing on particular objects or devices, it is the combination of technologies in use, and its relations to the systems in which it is embedded, that matters (Kanuka 2008). Understanding a VLE, for example, depends on local culture and infrastructure (Enriquez 2009). VLEs reinforce certain institutional roles and practices, and make others more difficult (Oliver 2011). Meanwhile, traditional practices and methods (e.g. lectures or tutorials) are perpetuated by community take-up of other technologies (Oliver 2011), such as Zoom or other videoconferencing software during the COVID-19 pandemic (Fox et al. 2021; Rapanta et al. 2020). Yet individual educators still have some agency to configure different approaches with most technologies (e.g. virtual writing retreats use silence to encourage participation in parallel activity via Zoom, see Koulaxi and Kong 2022).

#### Methods

Methods are structured templates for how teachers and students should proceed in the facilitation of learning. Examples include lectures, tutorials, problem-based learning, simulation and self-directed learning. Methods require technologies (e.g. classrooms, VLEs, videoconferencing software), yet can also, themselves, be understood *as* technologies, where they are used to reify particular ideas about learning or about how people should act (Dron 2021). Teaching methods convey values to students, whether implicitly or explicitly (Biesta 2010). Methods can become normative, imagined right ways of doing things within a particular context. They do not directly determine activity but function as guides to ‘acceptable kinds of action’ (Oliver 2011: 379). More rigid methods are, in theory, less reliant on skilful orchestration and are, therefore, more reliable than flexible methods (Dron 2021). Dron gives the example of a ‘rigid lesson plan’ as a ‘hard’ technology that shuts down possibilities for student activity.

However, methods do not simply ‘work’ by following a script, they must be enacted in accordance with values, purpose and the learning and teaching context (Biesta 2015). Furthermore, formal methods are only ever part of any student’s learning activity (Ellis and Goodyear 2009), and informal activities have a significant influence on learning during and after a course (Dron 2021). Situated teaching or learning activity, with its dynamic expressions of agency and discretion, often does not neatly fit strict criteria for what constitutes a method (Davis 2017).

#### Purposes

Making educational purposes explicit helps teachers and students to know not just what they will do but *why* (Kanuka 2008). However, teachers may hold only vague notions of purpose, and a clear articulation of purposes (particularly longer-term ones) is challenging (Priestley et al. 2015). As Priestly and colleagues note, superficial, short-term purposes (e.g. sessional outcomes) are problematic because they narrow possibilities for action and thus the agency of teachers and students. Learning outcomes are insufficient because much learning is emergent and, therefore, unpredictable (Fawns et al. 2021d), and there are always multiple purposes for any educational activity (Biesta 2009). Biesta argues that educational purposes can be located within three broad categories: qualification, socialisation and subjectification. Qualification involves the development of knowledge, skills and understandings that prepare students to contribute to economic growth, citizenship and forms of literacy necessary to function in society. Socialisation prepares students for ‘existing ways of doing and being’, to take on norms and values, and to become members of ‘social, cultural and political “orders”’ (p. 40). In contrast, subjectification prepares students to be autonomous and independent (e.g. through critical thinking or appreciation of diversity). Biesta argues that, whatever the intentions, education always contributes to all three, though these different purposes can be in tension.

Purposes must also be negotiated across stakeholders. Teachers may have additional purposes relating to their development, or to the production of work that will be useful in the future. Institutions have purposes relating to revenue, reputation, etc. Each student may hold multiple purposes for any learning task (e.g. achieving a good grade, learning how to do something, getting to know peers, learning to learn). An appreciation of purpose helps effective communication between stakeholders (Kanuka 2008) and, in quality education, purposes are carefully aligned with methods and values (Biesta et al. 2015; Kanuka 2008).

#### Values

Educational values are beliefs about what matters within learning and teaching (Fawns et al. 2022), including ideals, standards, principles and qualities of intrinsic worth (Collinson 2012). Dron (2021), for example, argues that ‘caring for the subject, for learning, and for the learner are non-negotiable’. Other examples include vulnerability (Lee 2021), collaboration (Fawns et al. 2021b) or critical thinking (Harland and Pickering 2010). Along with purpose, values underpin *why* educators do what they do (Biesta 2015). They influence what content, tasks, social groupings or forms of assessment are prioritised, and provide a basis for interpreting evidence about practice (Biesta 2010; House and Howe 1999). Values are inevitable in design, practice (Gudmundsdottir 1990) and evaluation (Biesta 2015), yet often remain implicit, especially where a discipline or culture emphasises objectivity and rationality (Harland and Pickering 2010). Apparently objective and standardised evaluation often involves default values of efficiency and effectiveness (Biesta 2009). Without clearly articulated values, educators are limited in their possibilities for action (Harland and Pickering 2010).

Values are also shaped through practice and context (Veugelers and Vedder 2003). *How* students learn is important (Biesta 2015; Feenberg 2006) because there is always collateral learning and side effects. Often, teachers must use their discretion to align educational practice with their values in shifting contexts (Fawns et al. 2021b), which can be difficult with inadequate materials, systems, teaching conditions or teaching expertise (Veugelers and Vedder 2003). As Priestley et al. (2015: 54) argue, values do not exist ‘in a vacuum but are themselves the result of the range of influences, demands and pressures that structure the settings – the particular ecologies – within which teachers think and act’. Choices of technology and method can, intentionally or otherwise, convey and promote certain values (Harland and Pickering 2010). For instance, a teacher may value trusting educational relationships whilst still requiring students to submit assessments to plagiarism detection software. Thus, values can remain aspirational (Fawns et al. 2021b) where educators have insufficient knowledge or agency to put them into practice. Indeed, ‘pedagogy first’ is suggestive of an aspiration towards teacher agency as a democratic value (Feenburg 2001).

#### Context

Being sensitive to context means taking account of information beyond what is in immediate focus, when making sense of complex activity (Korica and Nicolini 2019). This might include students’ personal histories, cultural backgrounds, home lives, studying conditions, goals, motivations, economic pressures, domain-specific considerations (e.g. the particular requirements of medicine, law or architecture), practical pressures (e.g. scheduling and resources) and so on, each of which is also complex in its own right. The contexts in which other stakeholders operate (e.g. administrators, policymakers, disciplinary bodies) are also relevant (Dron 2021; Fawns et al. 2021a). Yet, ‘context’ is a dangerous shorthand. It can include almost anything, potentially substituting for detailed analysis, and obscuring, rather than illuminating, important parts of the ‘wider picture’ that influence situated activity (Nicolini 2013: 234).

Just as they must identify relevant purposes and values, teachers must also decide which contextual elements are relevant, how, and why (Shulman 1986). For instance, rather than simply designating technologies as contextual elements, teachers can consider their specific historical relations with students and teachers (e.g. lectures or Zoom have accumulated cultural and personal baggage). Teachers must also account for how physical environments, materials and social arrangements are influenced not only by educational design but also by institutional policies and centralised configurations of technology (Goodyear and Carvalho 2019). What is relevant is not always knowable beforehand: context does not simply pre-exist learning activity, it is also shaped by it (Ellis and Goodyear 2009). Once a course begins, further contextual elements come into play, relating to the emergent co-configuration of students and teachers (Ellis and Goodyear 2009; Sun and Goodyear 2020).

## An Aspirational View of Entangled Pedagogy

The entangled model (Fig. 1) encourages educators to consider the diverse ways in which teachers and students actually engage with technology whilst learning, and how these are influenced by a range of situated factors (Jones 2015). In Column 3, the entangled elements are presented as non-hierarchical, acknowledging their mutual shaping (Tsui and Tavares 2021) and the value of a holistic, non-directional, non-linear view of relations (Chandler 1995). Each factor is no more or less important than any other. Combinations matter more than individual elements.

**Fig. 2:**
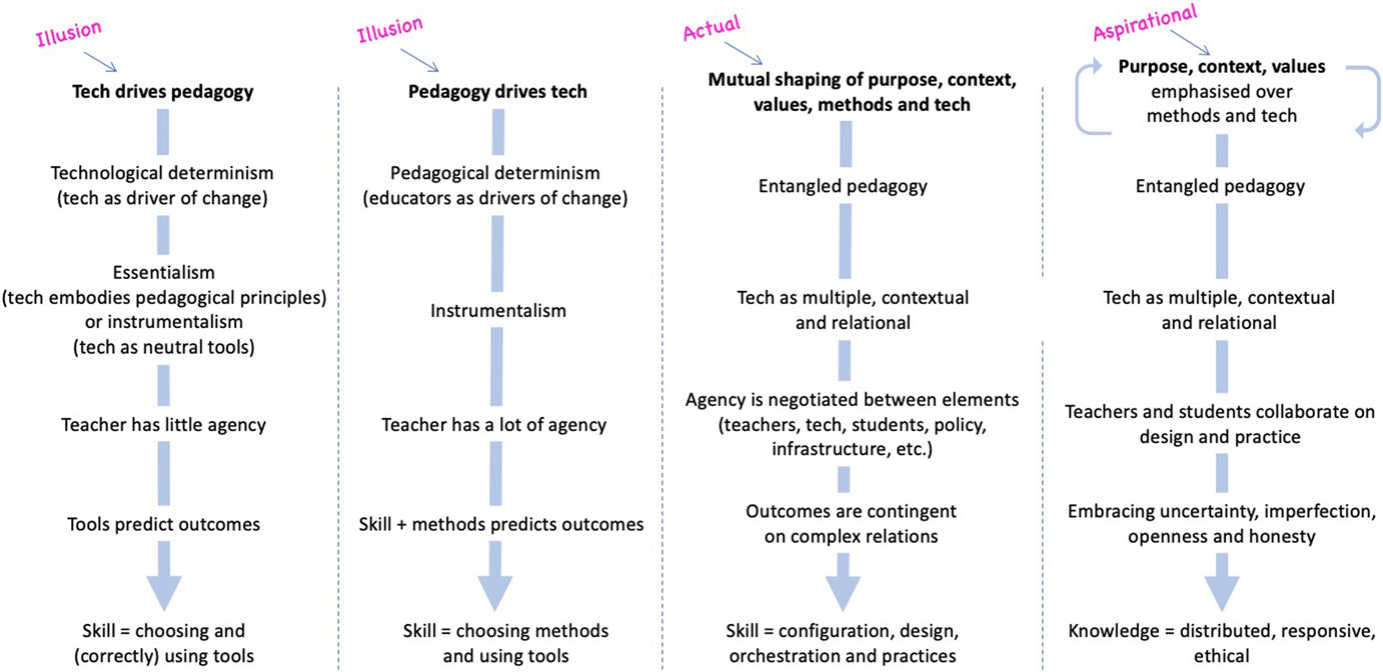
An entangled pedagogy, including an aspirational view

Nonetheless, the relative emphasis placed on each element by teachers, students and other stakeholders is worth attending to. In Fig. 2, I have added Column 4: an aspirational view of how teachers, students and other stakeholders can engage with the emergent complexity of educational activity. This view recognises that methods and technology tend to become over-emphasised in educational discourse, design and practice (Dron 2021). It suggests a need to intentionally and regularly revisit purposes, values and contexts, to ensure that they meaningfully and iteratively inform choices around methods and technology, whilst also recognising the shaping role of technology and methods as part of the pedagogical mix.

A ‘pedagogy first’ view may be an intentional effort to emphasise method, context, values or purpose over technology. However, in de-emphasising technology, it is important to recognise its inevitable entanglement in the mutual shaping of elements, particularly when we consider how teaching is entangled in institutional structures. For instance, values of academic integrity can be enacted, via technologies such as online proctoring, as surveillance and control (Fawns and Schaepkens 2022). IT staff, administrators and managers may not be aware of discrepancies between what teachers intend to value and what is actually valued in practice. Attending to values, purposes and context can help us identify problematic assumptions, such as those embedded in simple solutions to complex problems, reductive characterisations of students (e.g. as ‘digital natives’, see Oliver 2011), or assertions that teachers should conform to modern digital culture and practices (Clegg 2011).

A meaningful account of these interdependent factors requires a complex analysis that produces actionable knowledge (Markauskaite et al. 2020). This analysis should be based on observation, evidence and dialogue that focus on relations rather than individual elements (Goodyear and Carvalho 2019). Each stakeholder may hold different values and purposes, and have different contextual forces acting upon them. Since students co-configure designs, it makes sense to work with them to analyse their learning conditions, and to discuss these as part of the course (see Fawns et al. 2022 for an example). Ideally, this could inform collaborative design and orchestration of the course, as well as helping students to reflect on and reconfigure their learning environments. Including stakeholders such as administrators, learning technologists, or employers, in these discussions, could help to make the different elements more explicit and visible.

The negotiation of agency and the emergent outcomes produced through such complex entanglements suggest a need for trusting partnerships through which teachers, students and others can collaborate in educational activity. At course level, this may require teachers to embrace uncertainty, imperfection, openness and honesty, and to help students make sense of what is learned during a course, what must still be learned afterwards and how to go about it (Fawns et al. 2022; Fawns et al. 2021d). This is challenging, and teachers need support to continue their own learning. Clarifying purposes, contexts and values, and reconciling these with decisions about technology and methods, is easier when educators have expertise, discretion and confidence in the local culture and infrastructure which are, in turn, rooted in the institution’s valuing of educational expertise and pedagogy (Fawns et al. 2021c).

## Entangled Knowledge

Teaching with technology requires a non-trivial combination of different kinds of knowledge. Koehler et al. (2013) build on Shulman’s (1986; 1987) *pedagogical content knowledge* to propose the TPACK framework. This involves technological knowledge (how to work with information technology), pedagogical knowledge (ways of teaching and learning) and content knowledge (the subject matter to be learned). Different kinds of knowledge are considered in combination rather than individually. For example, computer literacy (technological knowledge) is important, but must be combined with pedagogical knowledge to encompass ethics, philosophy or the relationship between technology and learning (all of which constitute *technological pedagogical knowledge*) (Koehler et al. 2013). Recognising the need for thoughtful, nuanced negotiation of how different factors, and their combinations, shape educational situations, TPACK prioritises some aspects (purposes, values, learner characteristics) over others (techniques and methods).

Whilst TPACK is useful in establishing the broad forms of knowledge required by individual teachers, entangled pedagogy is collectively enacted. Teachers hold considerable responsibility, yet have only partial or ‘relational’ agency, and must work with others in negotiating outcomes (Edwards 2010: 61). Success is also reliant on students, who, in turn, operate within structures that are governed by teachers, institutions and regulatory bodies. Administrators, technical support staff, policymakers, employers, educational technology developers, etc. all contribute to the enactment of pedagogy, sometimes pulling in different directions. Centralised adoption of VLEs, for example, constrains possibilities for course-level practitioners (Feenberg 2006; Winner 1980).

Understanding this complexity allows teachers to attend holistically to situations (including advocating for change beyond course level), and to see how changes emerge slowly and indirectly, over time. However, educational activity will benefit most from the effective negotiation and distribution of knowledge combinations across stakeholders. The entangled pedagogy model can extend TPACK to consider the broader contexts in which teachers’ knowledge is situated (see also Mishra 2019 on including contextual knowledge within TPACK), and the knowledge required by those other than teachers. Different stakeholders have different levels and forms of expertise, and multiple strategies for supporting knowledge development are needed (Koh 2020), including involvement in dialogue, design, and application (Sharpe and Oliver 2013).

### Entangled Ethics and Ethical Knowledge

The combination of multiple technologies within an educational context always produces intended and unintended, predictable and unpredictable consequences and ‘side effects’ (Adams 2020; Chandler 1995; Dron 2021). The entanglement of technology is an ethical as well as a pedagogical issue, and evaluation should include potential harms (Fawns et al. 2021a). This involves going beyond functionality or implementation, to understanding how technologies work and the implications of their integration into specific contexts. Ethics requires more than following ‘a linear chain of events’ (Barad 2007: 384); it means tracing relations to see where they lead.

Here, it is important to differentiate between ethical values (e.g. inclusivity, fairness) and the required knowledge to embed them within practice. Whilst values (including, but not limited to, ethical values) inform the ‘actual’ view (Column 3) of entangled pedagogy, complex ethical knowledge is part of the ‘aspirational’ view (Column 4). This recognises the challenge before us. Ethics in technology and education is under-researched and poorly understood (Moore and Ellsworth 2014). Although ethics is briefly acknowledged within the TPACK framework, further clarification of the required ethical knowledge is needed (Adams 2020; Asamoah 2019) along with how it might be distributed across stakeholders (Dron 2021).

At course level, we can consider how students’ home spaces, physical devices, broadband and infrastructure shape the ways in which they are able to engage with tasks, teachers and peers through digital technology (Fawns et al. 2022). We can ask how social and material designs combine with digital interfaces to mould experiences of typing, watching, speaking or sitting; how they constrain physical movements or the configuration of workspaces (e.g. being on camera might encourage a student to sit in a particular location within the home that looks more presentable); or how they shape or reinforce power relations and social dynamics (e.g. by reinforcing differences in appearance, wealth or disposition). At an institutional level, there are ethical implications of how technology is embedded within infrastructures and policy (Williamson and Hogan 2021). Amongst other things, we need to know how data are stored, interpreted and used (e.g. what access and control do students have over their data), how stable platforms will be over time (e.g. might they be discontinued; will their terms and conditions change) and how students are affected by configurations (e.g. requiring students to submit work to plagiarism detection software affects trust relations [Ross and Macleod 2018]).

Like pedagogy, ethics cannot be the sole responsibility of teachers. Working relationships between University IT staff and commercial providers shape not only broader institutional implementations of technology, but the distribution of pedagogical and ethical knowledge and decision-making. The higher-level configurations of VLEs, learning analytics interfaces, videoconferencing software, etc. should be understood by members across educational institutions, not just in terms of economics, legality, data security or technical support, but also in ethical and pedagogical terms (Williamson 2016). Just as teachers need more than pedagogical *or* technological knowledge in isolation but a combination of those forms of knowledge, so too do those with responsibilities in procurement, support, maintenance, implementation, policy-making and faculty development.

We should ask ethical questions, not only about new technology but about all technology. Though the increasing integration of digital platforms and devices makes these issues more pressing, the entanglement of technology and pedagogy in education is not a new phenomenon (Cousin 2005; Murphy et al. 2001). For this reason, Adams (2020) problematises the idea of technological knowledge, arguing that when it ‘overlaps with other knowledges, [it] will ultimately become transparent and thus slip from attention’ (p. 53) as the relevant technology becomes more integral within teaching practice. In other words, technology becomes context, peripheral to material or embodied considerations of design and practice (Gourlay 2021). When faced with the unfamiliarity of the new, or the invisibility of the old, ignorance of the ethics of technology is not an excuse: we are always part of entanglements, and we are always partly responsible for them (Barad 2007).

## Implications for Evaluation and Research

Research based on views represented by Columns 1 or 2 of the entangled model is potentially misleading. For example, media comparison studies (e.g. Zoom vs physical classroom, or online vs on campus) are often underpinned by deterministic views. Outcomes are seen as products of technology, method or modality, yet variance within conditions is often greater than variance between them (Dron 2021; Saba 2000), and statistical tests are confounded by the complex interrelations shown in Column 3. Problematic simplifications are used to produce clear results (Shulman 1987), but researchers often find no significant differences (Dron 2021; Lockee et al. 2001; Saba 2000). As Saba (2000) argued, over 20 years ago, this stems from the lack of explanatory theoretical frameworks to make results relevant to other researchers and practitioners.

Moreover, benefits of education are largely contingent on localised purposes and values (Kanuka 2008). Many recent studies describe digital learning environments (particularly in online or hybrid learning contexts) as independent of their social and material contexts (Fawns 2019; Gourlay 2021). The entangled pedagogy model is agnostic about modality (whether a course or programme is on campus, online or hybrid), because the relationship of technology to the situated combination of context, purposes, values and methods is more important. Whilst there are important differences between online, on campus and hybrid teaching, those labels are insufficient representations of the interplay of elements. Digital education is always also material, social and embodied (Fawns et al. 2019), and there is always some digital aspect of any educational activity in contemporary higher education, irrespective of modality. Looking past modality, and crude categories (e.g. ‘Zoom’ or ‘lectures’), allows us to contextualise what is actually happening and respond to the diverse and situated needs in front of us. Technologies and methods are always emergent assemblages of material, social and digital activity (Fawns 2019; Fenwick 2015), albeit with features and patterns of commonality (Goodyear 2021).

The unit of analysis for entangled evaluation is combinations rather than components. Neither technologies nor teaching methods can be evaluated in isolation of the contexts in which they are embedded (Dron 2021; Fawns et al. 2021a; Fawns and Sinclair 2021). Each element in Column 3 is also internally entangled. Technologies are part of the educational context, but can also be objects of study (i.e. part of educational purpose). Methods can be understood as technologies; understanding technology in use involves interrogating values, which are shaped by context, and so on. Thus, during the pedagogical dance, the dancers (purpose, context, values, methods and technology) are in a constant whirr of motion, defying separation, just as only white can be seen in a spinning wheel of colours. It is impossible to put technology or pedagogy first or last because each element must be understood as integrated within a greater, emergent entanglement that has no clear beginning or end (Barad 2007). Entangled elements (or, as Barad calls them, ‘agencies’) co-constitute each other: there is no method without technology, no values without context, and so forth. Pedagogical entanglements are fluid and ‘highly specific configurations’ (p. 74). Thus, outcomes are not caused by technology, methods or teachers, but entanglements, and claims must be made ‘in relation to the whole phenomenon, and not to elements … taken out of context’ (Oliver 2011: 377).

Rather than assuming that certain approaches (e.g. remotely invigilated exams, synchronous video-conferenced tutorials or assessment of discussion forums to disincentivise ‘lurking’) will result in particular outcomes for every student, educators might take account of how policies, processes, personal circumstances, study environments and so on can make some activities problematic and, potentially, further increase the drive for students to subvert expectations (Fawns and O’Shea 2019). In relation to both direct and side effects, as well as the ethical entanglements described above, educators can also consider how particular integrations of technology in education align with their values, and with the values of their students (Adams 2020; Dron 2021).

## Conclusions

Whilst calls to put pedagogy first are understandable, they misrepresent the ways in which technology inevitably shapes teaching and learning activity, and may lead to inadequate appreciation of the possibilities, constraints and risks of technology in education. Nor can technology be the sole driver of pedagogy. Proposals that technology can solve educational problems, or produce particular outcomes, oversimply the ways in which technology is embedded in context (Fawns 2019). Discussions of pedagogy driving technology, or vice versa, reinforce a false dichotomy. Pedagogy is not just method, and technology is not just a vehicle for implementing that method. Pedagogy involves the negotiation of methods and technologies, in relation to purposes and values, in a subjectively understood context.

In this paper, I have presented a model of entangled pedagogy, in which technology is one of a number of elements that are always interdependent *within* complex pedagogical activity. This model provides a stronger basis for making choices that align with educators’ values, purposes and contexts, as well as with those of their students. It considers three different ways of understanding the relationship between pedagogy and technology (technology first, pedagogy first and entangled), before proposing an aspirational view of how educators can meaningfully take account of the complexity of these relationships in design and practice. Here, purpose values and contexts are emphasised by continually revisiting them during teaching design and orchestration. At the same time, teachers collaborate with students and others to collectively negotiate these factors, and pedagogical knowledge is distributed across stakeholders at different levels of the institution (Dron 2021). This is important not only for the effectiveness but also the ethics of any entangled educational activity.
